# The selection of an optimal transportation strategy in urgent stroke missions: a simulation study

**DOI:** 10.1186/s13049-020-00747-4

**Published:** 2020-06-01

**Authors:** Jukka Pappinen, Tuuli Miettinen, Päivi Laukkanen-Nevala, Pekka Jäkälä, Anne-Mari Kantanen, Pekka Mäntyselkä, Jouni Kurola

**Affiliations:** 1 FinnHEMS Research and Development Unit, Lentäjäntie 3, FI-01530 Vantaa, Finland; 2 University of Eastern Finland, Faculty of Health Sciences, P.O. Box 1627, FI-70211 Kuopio, Finland; 3 University of Eastern Finland, Institute of Clinical Medicine, Unit of Neurology, Kuopio, Finland; 4 Neuro Center, Kuopio University Hospital, P.O.B. 100, FI-70029 KYS Kuopio, Finland; 5 University of Eastern Finland, School of Medicine, P.O. Box 1627, FI-70211 Kuopio, Finland; 6 Primary Health Care Unit, Kuopio University Hospital, Kuopio, Finland; 7 Centre for Pre-hospital Emergency Care, Kuopio University Hospital, P.O. Box 1777, FI-70210 Kuopio, Finland

**Keywords:** Emergency medical services, Geographic information systems, HEMS, Stroke, Simulation

## Abstract

**Background:**

Stroke causes death, disability and increases the use of healthcare resources worldwide. The outcome of intravenous thrombolysis and mechanical endovascular thrombectomy highly depends on the delay from symptom onset to initiation of definitive treatment. The purpose of this study was to compare the various patient transportation strategies to minimize pre-hospital delays.

**Methods:**

Emergency medical services (EMS) mission locations and ambulance response times in Finland with urgent stroke-suspected dispatch codes were collected from Emergency Response Centre (ERC) records between 1 January 2016 and 31 December 2016. Four transport scenarios were simulated for each mission, comparing ground and helicopter transportation to hospital with different treatment capabilities.

**Results:**

In 2016, a total of 20,513 urgent stroke-suspected missions occurred in Finland. Of these, we were able to locate and calculate a route to scenario-based hospitals in 98.7% (20,240) of the missions.

For ground transport, the estimated median pre-hospital time to a thrombolysis-capable and thrombectomy-capable hospital were 54.5 min (95% confidence interval (CI), 31.7–111.4) and 94.4 min (95% CI, 33.3–195.8), respectively. Should patients be transported on the ground to thrombectomy-capable hospitals only, the pre-hospital time would increase in 11,003 (54.4%) of missions, most of which were in rural areas.

With the fastest possible transportation method, the estimated mean transport time to a thrombectomy-capable hospital was 80.84 min (median, 80.80 min; 95% CI, 33.3–143.1). Helicopter transportation was the fastest method in 68.8% (13,921) of missions, and the time saved was greater than 30 min in 27.1% (5475) of missions. In rural areas, helicopter transportation was the fastest option in nearly all missions if dispatched simultaneously with ground ambulance.

**Conclusion:**

Helicopter transportation may significantly decrease pre-hospital delays for stroke patients, especially in rural areas, but the selection of an optimal transportation method or chain of methods should be determined case-by-case.

## Introduction

Acute ischemic stroke is the second most common cause of death worldwide [1] and results in disability for most patients and strain for healthcare services. In 2014, there were 20,467 new stroke events in Finland, which resulted in 201,892 patient days in a hospital ward. In 2007, the total costs of stroke patient care in Finland was approximately $1.6 billion, which was approximately 7% of total health care expenditure [2].

Intravenous thrombolysis with tissue plasminogen activator (tPA) within 4.5 h after symptom onset is standard care for acute ischemic stroke. Recently, several randomized control trials also demonstrated the efficacy and safety of intra-arterial mechanical treatment [3–7]. In 2018, two studies were published that showed that thrombectomy for acute ischemic stroke was effective for up to 16 and 24 h among selected patients [8, 9]. Although studies have shown the efficacy, safety, and effectiveness of thrombectomy even after relatively long durations since symptom onset, the probability of good outcome is directly related to delay from symptom onset to reperfusion [10–12]. Thus, shortening the duration of the total process should be the main goal when developing acute stroke care system.

In most countries with organized acute healthcare services, equity in access to healthcare services is valued [13]. Several studies have focused on measuring, analysing, and shortening emergency medical services (EMS) response times [14–17]. Similarly, the current legislation in Finland demands that hospital districts determine, follow, and report EMS response times. In Finland, patients’ travel times from home to hospital were previously evaluated [18], but the total time from an emergency call to the hospital (i.e., the pre-hospital time) has not been systematically evaluated, despite its relevance to the outcome of treatment.

Helicopter emergency medical services (HEMS) has been shown to effectively shorten pre-hospital times in certain patient groups [19], although the benefit highly depends on the general structure of EMS and the hospitals’ location. The HEMS base location has been suggested as one mechanism to diminish inequity in access to emergency care, especially for trauma patients [20, 21].

This study aimed to 1) compare strategies for stroke patient transportation to determine an optimal strategy to minimize pre-hospital time and 2) report predicted interregional and urban-rural variations in delays in access to thrombolysis- or thrombectomy-capable hospitals. The study focuses on operational and logistics issues; thus, no economical evaluation is performed.

## Methods

The Finnish Emergency Response Centre (ERC) is a state agency that is responsible for handling calls made to the public emergency number, 112. The ERC handles requests for EMS, police, fire and rescue, and emergency social services. Currently, there are six national ERCs to where 112 calls are routed based on the callers’ geographic locations.

In EMS-related calls, dispatchers use a computer-aided and criteria-based risk assessment system to classify missions to one of four urgency classes (i.e., A-D) and 83 symptom and findings-based or injury mechanism-based classes. Dispatchers do not attempt to identify the patient, nor do they have access to electronic patient records. Legally, the caller or victim is not a patient until he or she is reached by an EMS unit. Every call is registered in ERC records.

EMS mission data between 1 January 2016 and 31 December 2016 with urgent suspected-stroke dispatch codes were collected from ERC records. In this study, we analysed only the mission and urgency class code, mission location, and response time of the first ambulance unit. The patients were not identified, nor were patients’ hospital or EMS records accessed. The incident location and ground ambulance response time data were combined with the estimated transportation time to determine the time delay from an emergency call to patient arrival to the nearest hospital with stroke thrombolysis or thrombectomy capabilities. In Finland, only university hospitals (*n* = 5) have thrombectomy capability. Currently, the suspected-stoke criteria-based risk assessment tool used in ERCs is not designed or able to identify potential thrombolysis or thrombectomy candidates, and therefore, missions are mostly over-triaged [22].

We estimated the pre-hospital time for four scenarios for each mission:

- Direct ground ambulance transportation to the nearest hospital with computed tomography (CT) and thrombolysis capability (regional, central, or university hospital)
- Direct ground ambulance transportation to the nearest hospital with thrombectomy capability (university hospital)
- Helicopter transportation to the nearest university hospital with simultaneous ground EMS dispatch
- Helicopter transportation to the nearest university hospital with HEMS dispatched by EMS after primary evaluation

The catchment area for each hospital was determined by calculating a convex hull around a set of mission locations with the fastest road access to the same hospital. In this study, catchment areas did not align with administrative areas.

The area type for a mission scene was determined for each mission using the existing area classification method. Missions were divided into four groups by their area type: core urban, other urban, dispersed settlement, and other rural [23].

The open source GraphHopper [24] routing tool and OpenStreetMap [25] data were used to determine normal driving times, which were decreased by 20% to simulate a drive with the lights and siren based on literature [26]. Flight times were calculated via the great circle route and a flight speed of 220 km/h. Based on FinnHEMS and EMS statistics, we used 6 min as the dispatch to en-route time for a helicopter and 26 min as the on-scene time for both ambulances and helicopters. The patient unload time target at the hospital was 10 min; however, it is known that this time varies among hospitals depending on the helipad location, and accurate data is not available.

The results were reported as median times with a 95% confidence interval (CI). To support operative decision making with thrombectomy candidates, the effect of the estimated ground transportation time on the optimal transportation method was also reported.

## Results

In 2016, ERCs dispatched 20,513 urgent stroke missions. We were able to locate and calculate the routes to the hospitals in 20,240 (98.7%) missions, which were included in the study.

With ground transportation only, the estimated median pre-hospital time to thrombolysis-capable and thrombectomy-capable hospitals was 54.5 min (95% CI, 31.7–111.4) and 94.4 min (95% CI, 33.3–195.8), respectively.

If the patients were transported only to thrombectomy-capable hospitals, the pre-hospital time would increase in 11,003 (54.4%) missions. The median increase was 66.8 min (95% CI, 8.5–144.0). In rural areas, the pre-hospital time would increase in roughly 70% of the missions (Table 1).

**Table 1:** The total pre-hospital time using ground transportation only in the simulation

		Total pre-hospital time with ground transportation (min)		Additional delay if transported directly to a thrombectomy-capable hospital	
		Thrombolysis-capable hospital	Thrombectomy-capable hospital		
	n	median (95% CI)	median (95% CI)	N (%)	median (95% CI)
Core urban	4796	32.6 (29.3–80.5)	78.4 (30.5–177.7)	2410 (50.3)	76.1 (16.4–144.6)
Other urban	12,305	54.8 (34.4–106.0)	89.8 (36.8–193.6)	6434 (52.3)	65.9 (7.6–144.0)
Dispersed settlement	2852	84.3 (51.9–134.7)	127.0 (64.3–219.6)	1948 (68.3)	63.8 (5.8–144.0)
Other rural	287	96.3 (54.1–166.9)	148.3 (69.7–257.6)	211 (73.5)	65.1 (7.7–144.0)
**Total**	**20,240**	**54.5 (31.7–111.4)**	**94.4 (33.3–195.8)**	**11,003 (54.4)**	**66.8 (8.5–144.0)**

Helicopter transportation to a thrombectomy-capable hospital was faster in 68.8% (13,921) of the missions, with a median time benefit of 23.7 min (95% CI, 2.3–77.1), if HEMS was dispatched simultaneously with ground EMS (Table 2a). If HEMS was dispatched by paramedics after patient evaluation, the time benefit decreased. However, helicopter transportation to a thrombectomy-capable hospital would have been faster than ground transportation in 41.9% (8483) of missions, with a median time benefit of 16.9 min (Table 2b). In rural areas, helicopter transportation would be the fastest option in nearly all missions if dispatched simultaneously with a ground ambulance.

**Table 2:** The pre-hospital time saved by helicopter transportation compared to ground transportation

a)	Simultaneous dispatch		
	N (%)	Median (95% CI)	> 30 min saved (%)
Core urban	2551 (53.2)	19.1 (1.8–58.4)	635 (13.2)
Other urban	8303 (67.5)	21.6 (1.9–64.5)	3037 (24.7)
Dispersed settlement	2784 (97.6)	34.2 (6.4–92.4)	1590 (55.8)
Other rural	283 (98.6)	47.9 (10.8–141.7)	213 (74.2)
**Total**	**13,921 (68.8)**	**23.7 (2.3–77.1)**	**5475 (27.1)**
b)	dispatch after primary evaluation		
	N (%)	median (95% CI)	> 30 min saved (%)
Core urban	1546 (32.2)	13.3 (1.1–75.3)	283 (5.9)
Other urban	4819 (39.2)	17.7 (1.9–76.3)	1286 (10.5)
Dispersed settlement	1885 (66.1)	18.6 (1.4–85.7)	545 (19.1)
Other rural	233 (81.2)	24.0 (3.0–110.9)	97 (33.8)
**Total**	**8483 (41.9)**	**16.9 (1.6–75.7)**	**2211 (10.9)**

With the fastest possible transportation method (i.e., ground EMS or HEMS), the estimated mean time to a thrombectomy-capable hospital was 80.84 min (median, 80.80; 95% CI, 33.3–143.1). HEMS transportation was the fastest method in 68.8% (13,921) of missions, and the time saved was greater than 30 min in 27.1% (5475) of missions (Table 2a).

**Fig. 1:**
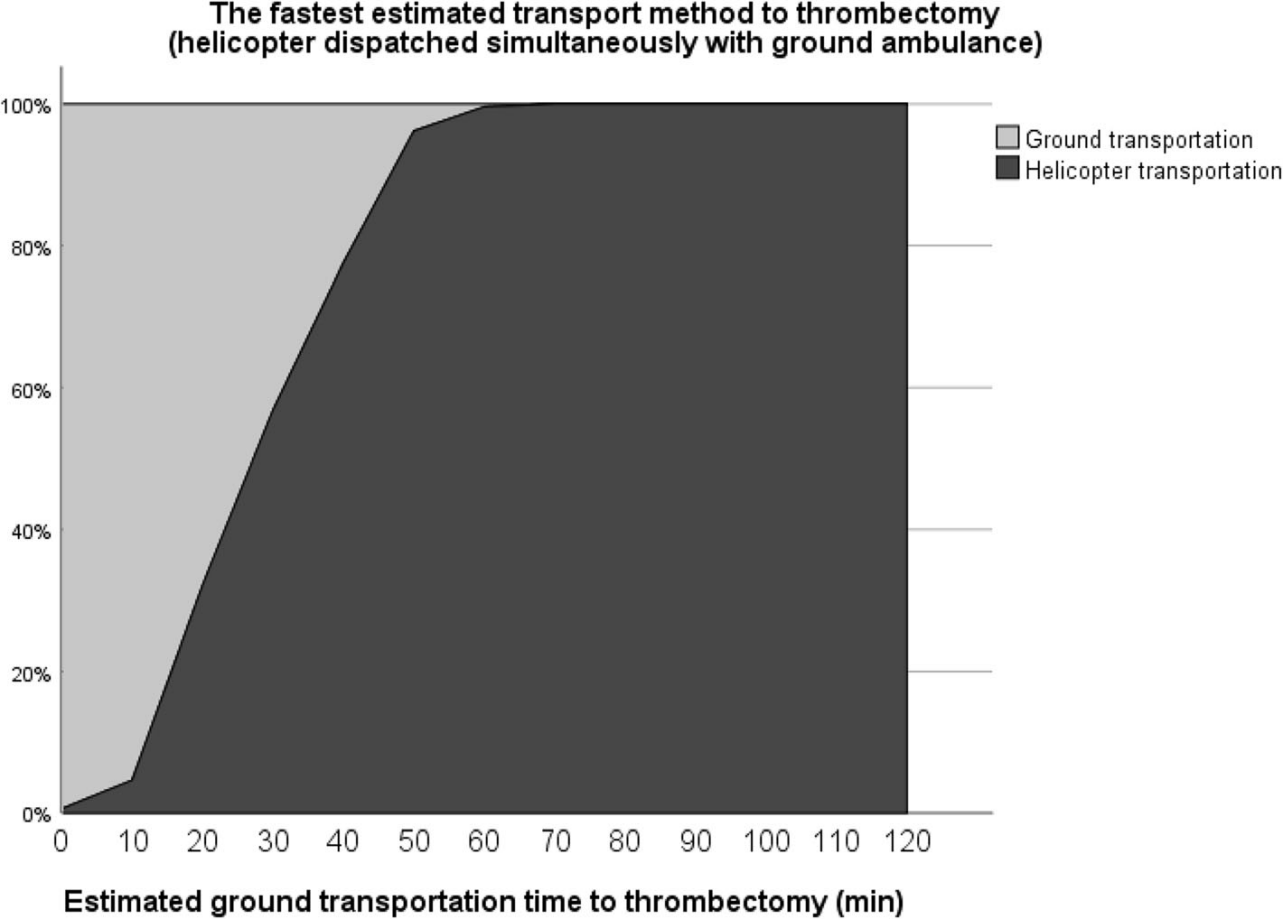
The fastest estimated transportation method to thrombectomy when HEMS is dispatched by ground ambulance crew

**Fig. 2:**
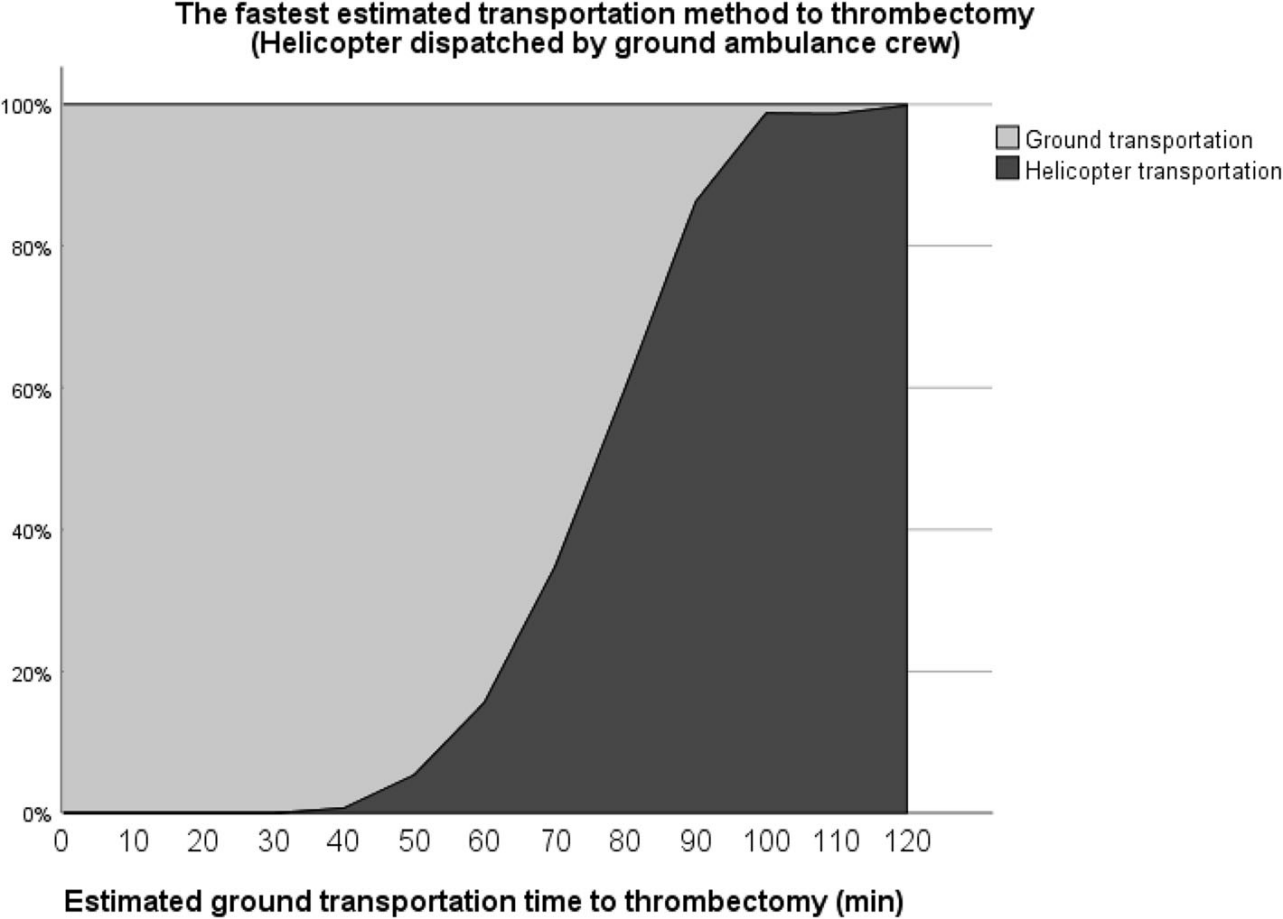
The fastest estimated transportation method to thrombectomy with simultaneous HEMS and ground ambulance dispatch

Helicopter transportation is increasingly becoming the optimal choice when ground transportation time increases. Should the ground ambulance crew dispatch HEMS after primary evaluation, helicopter transportation was estimated to be the fastest option in most cases with greater than 80 min ground transportation time (Fig. 1). If HEMS is dispatched simultaneously with a ground ambulance, helicopter transportation is the fastest choice in cases where estimated ground transport time exceeds 40 min (Fig. 2).

A supplementary thematic map shows a nationwide distribution of stroke-suspected missions and the fastest transportation methods (Supplementary file 1).

## Discussion

The main finding of this study indicates that utilization of helicopter transportation in time-critical conditions could diminish the total pre-hospital time, improve access to emergency care, and increase the equality between rural and urban areas. In stroke patients, access to thrombectomy could be significantly improved via helicopter transportation.

Currently, ERC dispatchers over-triage stroke-suspected missions, which results inefficient EMS resource use. Most patients with urgent stroke-suspected codes are not thrombolysis or thrombectomy candidates [22]. However, it is not possible to identify potential patients more accurately with the data available. To effectively use helicopter transportation, which is relatively expensive, ERC dispatchers’ ability to recognize symptoms of stroke and preferably identify those with major artery occlusion that would thus benefit from thrombectomy needs to be improved. For example, real-time video calls via mobile phone might offer a usable tool to evaluate the condition of a stroke patient.

Another problem is the selection of the receiving hospital. Typically, the nearest hospital has at least thrombolysis capability, but thrombectomy-capable hospitals are often further away. The length of additional transportation delays that are acceptable to provide better treatment capabilities is unknown. Door-to-needle time variations among hospitals should be considered in models to improve specificity; however, such benchmarking data is not yet available.

The transportation of a patient to the nearest hospital for imaging and, if indicated, the initiation of intravenous thrombolysis treatment that continues during secondary transport to a thrombectomy-capable hospital (i.e., the drip and ship strategy) may offer faster diagnosis and treatment initiation for certain patient groups. However, the outcomes are dependent on out-of-hospital and in-hospital processes and transportation delays.

The analysis suggests that the balance between optimal transportation methods is somewhat labile. The effect of relatively small changes in assumptions, driving times, or unit locations on mission onset may change the optimum transportation method and the optimum receiving hospital. The time to transport a patient from a hospital helipad to an emergency room (ER) may negate the time benefit from faster helicopter transportation. The options are often dichotomous and/or limiting and choices in one mission might alter the available choices in simultaneous missions or the next mission in the same area. Machine-learning systems might offer a reasonable method to estimate the outcomes of different options and support decision-making in real-time.

To use methods presented in this study to support decision-making in everyday practice, both pre-hospital and in-hospital delays in every phase of the logistic chain should be studied with comprehensive and accurate data collection. An economic evaluation should be performed to further evaluate costs and benefits of different transport strategies. Due to constantly changing unit locations, weather or road conditions, pre-calculated response time maps, and plans may lead to the selection of non-optimal transportation methods. A real-time decision support system utilizing current unit location, weather, and traffic data would be beneficial for decision-making during individual missions.

## Conclusions

Helicopter transportation may significantly decrease stroke patients’ pre-hospital time, especially in rural areas, but the selection of an optimal transportation method or chain of methods should be determined case-by-case.

## Supplementary information

**Additional file 1.** A thematic map showing the distribution of stroke-suspected missions in 2016 in Finland with the fastest estimated transportation method indicated.

## Data Availability

Anonymized data are available upon reasonable request from the correspondence author (https://orcid.org/0000-0002-1174-8669).

## Abbreviations

HD: Hospital District
EMS: Emergency Medical Services
HEMS: Helicopter Emergency Medical Services
ERC: Emergency Response Centre
ER: Emergency Room
