# Supplementary figures and images for: The selection of an optimal transportation strategy in urgent stroke missions: a simulation study
Source: Scand J Trauma Resusc Emerg Med. 2020 Jun 1;28:48. doi: 10.1186/s13049-020-00747-4 (PMC7268427; doi:10.1186/s13049-020-00747-4)

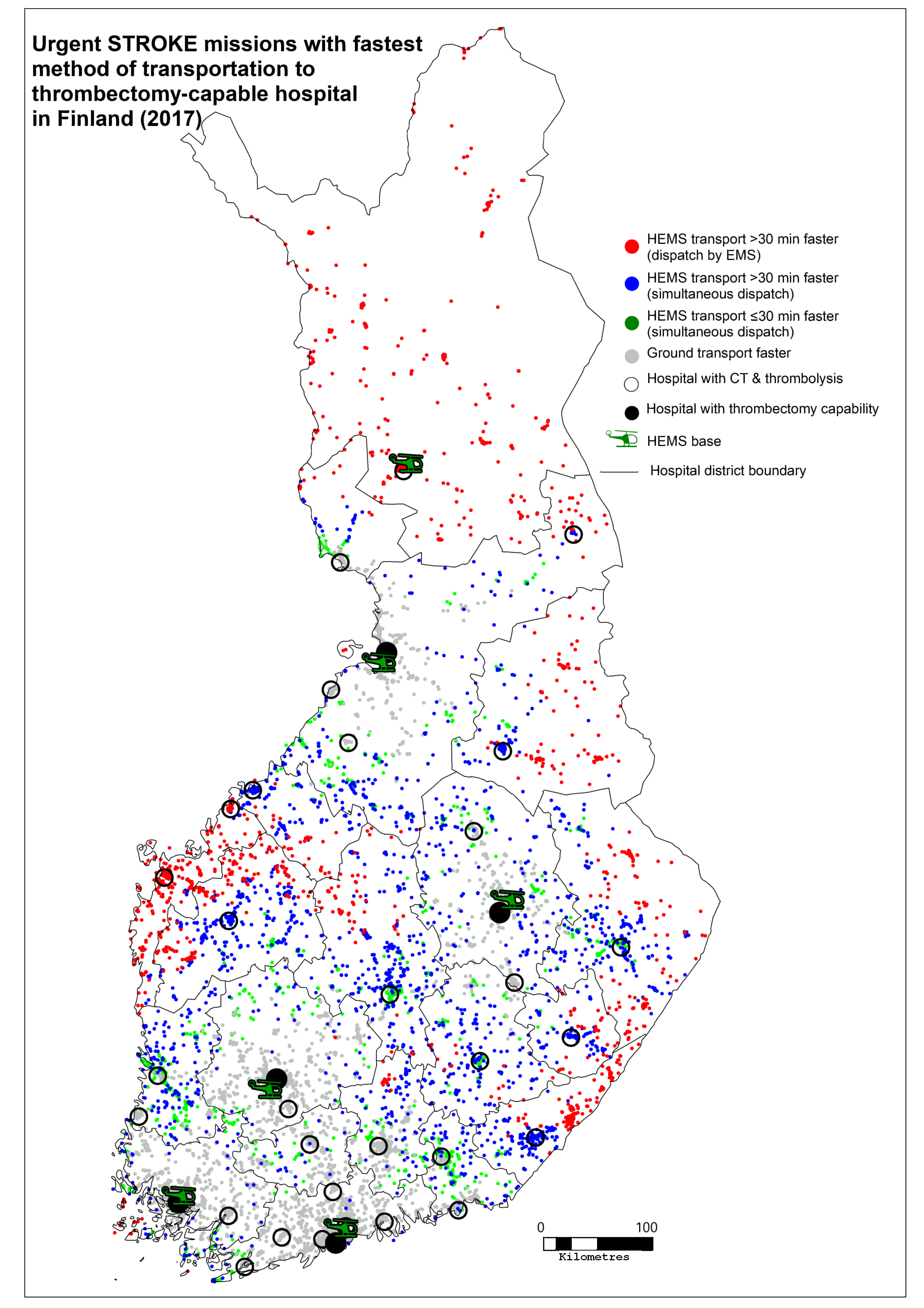

Supplement: Supplementary file 1 — Additional file 1. A thematic map showing the distribution of stroke-suspected missions in 2016 in Finland with the fastest estimated transportation method indicated. [file 13049_2020_747_MOESM1_ESM.tif]
